# Derivation and validation of a search algorithm to retrospectively identify mechanical ventilation initiation in the intensive care unit

**DOI:** 10.1186/1472-6947-14-55

**Published:** 2014-06-25

**Authors:** Nathan J Smischney, Venu M Velagapudi, James A Onigkeit, Brian W Pickering, Vitaly Herasevich, Rahul Kashyap

**Affiliations:** 1 Department of Anesthesiology, Mayo Clinic, 200 First St SW, Rochester, MN 55905, USA; 2 Multidisciplinary Epidemiology and Translational Research in Intensive Care (METRIC), Mayo Clinic, Rochester, Minnesota, USA

**Keywords:** Airway management, Electronic health records, Intensive care units, Mechanical ventilation initiation, Search algorithm

## Abstract

**Background:**

The development and validation of automated electronic medical record (EMR) search strategies are important for establishing the timing of mechanical ventilation initiation in the intensive care unit (ICU).

Thus, we sought to develop and validate an automated EMR search algorithm (strategy) for time zero, the moment of mechanical ventilation initiation in the critically ill patient.

**Methods:**

The EMR search algorithm was developed on the basis of several mechanical ventilation parameters, with the final parameter being positive end-expiratory pressure (PEEP), and was applied to a comprehensive institutional EMR database. The search algorithm was derived from a secondary retrospective analysis of a subset of 450 patients from a cohort of 2,684 patients admitted to a medical ICU and a surgical ICU from January 1, 2010, through December 31, 2011. It was then validated in an independent subset of 450 patients from the same period. The overall percent of agreement between our search algorithm and a comprehensive manual medical record review in the derivation and validation subsets, using peak inspiratory pressure (PIP) as the reference standard, was compared to assess timing of mechanical ventilation initiation.

**Results:**

In the derivation subset, the automated electronic search strategy achieved an 87% (κ = 0.87) perfect agreement, with 94% agreement to within one minute. In validating this search algorithm, perfect agreement was found in 92% (κ = 0.92) of patients, with 99% agreement occurring within one minute.

**Conclusions:**

The use of an electronic search strategy resulted in highly accurate extraction of mechanical ventilation initiation in the ICU. The search algorithm of mechanical ventilation initiation is highly efficient and reliable and can facilitate both clinical research and patient care management in a timely manner.

## Background

The present article is a subsequent report to an initial publication describing the programmatic processes used for developing and validating an automated electronic medical record (EMR) search strategy for identifying emergent endotracheal intubations in a medical intensive care unit (ICU) or surgical ICU [1]. In the initial publication, we used an automated electronic note search strategy for identifying patients who had emergent endotracheal intubation in the critical care setting [1]. The automated search strategy was a necessary first step and has not been explored previously. With this information, we now turn to the development and validation of an automated EMR search strategy to identify the time point at which mechanical ventilation began. The ability to accurately identify the temporal occurrence of a major clinical event (such as emergent endotracheal intubation) from the EMR allows, in combination with other clinically relevant parameters (e.g., heart rate, blood pressure, cardiac index, etc.), the in-depth retrospective analysis of factors which may help in the early identification of the deteriorating patient and the possibility to prevent adverse clinical events (such as post-intubation hemodynamic instability) in the future. Therefore, both these steps are needed before retrospectively evaluating possible risk factors associated with hemodynamic disturbances during emergent endotracheal intubations in critically ill patients.

Within an EMR, retrospectively identifying when mechanical ventilation was initiated requires considerable time and effort with manual medical record review. Several studies have assessed complications of emergent endotracheal intubation in the ICU [2-5]. However, these studies were prospective in nature, and therefore the initiation of mechanical ventilation was known. Establishing the timing of mechanical ventilation becomes more problematic when it is reviewed retrospectively. However, development and validation of an electronic search strategy may allow rapid and accurate timing of mechanical ventilation initiation in an emergent situation within the critical care setting when analyzed retrospectively.

Reports of electronic search strategies have increased in frequency, in large part because of the adoption of EMRs. Automated search strategies have shown high sensitivity and high specificity in recognizing various medical entities through an electronic surveillance system [6,7]. In addition, the search strategies have been used to identify risk factors for various clinical conditions and have resulted in highly accurate and efficient data extraction within an EMR [8]. Given recent evidence suggesting reduced medical costs and, possibly, enhanced patient safety with use of EMRs [9], reports of search strategies are likely to be published more frequently in the medical literature. With EMR implementation, various institutions have developed data warehouses for several quality improvement outcomes [10,11]. Therefore, electronic search strategies provide a potential solution to highly efficient data extraction within an electronic medical environment and allow rapid navigation of large databases.

Our primary aim was to develop and validate an automatic EMR search strategy to identify the timing of mechanical ventilation initiation in the critical care environment. Our secondary aim was to compare the overall agreement of an automatic EMR search strategy with a comprehensive manual medical record review (the reference standard).

## Methods

The study was approved by Mayo Clinic Institutional Review Board for the use of existing medical records of patients who gave prior research authorization.

### Study population

**Figure 1:**
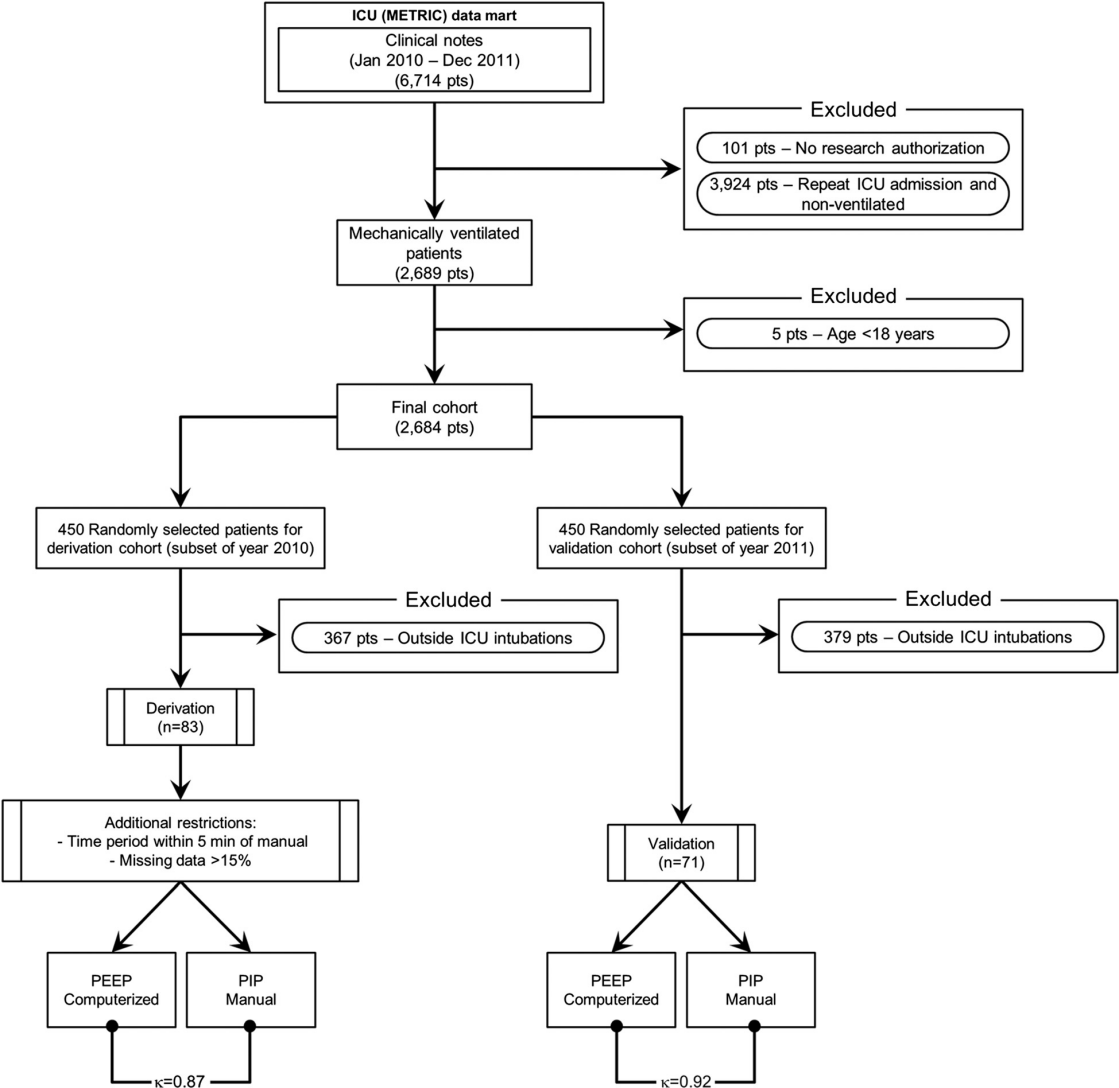
**Electronic search strategy flow diagram of patients in the ICU (METRIC) data mart from January 1, 2010, through December 31, 2011.** ICU indicates intensive care unit; METRIC, multidisciplinary epidemiology and translational research in intensive care; PEEP, positive end-expiratory pressure; PIP, peak inspiratory pressure; pts, patients.

The derivation and validation subsets were obtained retrospectively from two critical care units at Mayo Clinic in Rochester, Minnesota. This heterogeneous population of patients in the medical and surgical ICUs was admitted from January 1, 2010, through December 31, 2011. The total cohort included 6,714 patients. The cohort was reduced to contain only the 2,689 patients who received both invasive and non-invasive mechanical ventilation on their first ICU admission during the study period, excluding 101 patients who did not provide prior research authorization (Figure 1). From this cohort, five patients were excluded because of age restriction (<18 years). A subset of this cohort was used for derivation and consisted of 450 randomly selected patients treated in 2010. This group was further reduced to 83 patients who, with the same criteria, were intubated emergently in the ICU (invasive). The search algorithm was then validated against 450 randomly selected patients treated in 2011, and subsequently reduced to 71 patients with the same criteria from the above-mentioned cohort using JMP statistical software (version 9.0; SAS Institute Inc). The selection of 450 patients for the derivation and validation cohorts was made to minimize the burden for the investigators during manual medical record review while ensuring a robust sample size for both subsets. We used the search algorithm published previously for identification of emergent endotracheal intubations [1].

### Manual data extraction strategies (Reference Standard)

The medical records of the derivation and validation groups were manually reviewed by two independent critical care clinicians (N.J.S and V.M.V). Each record was evaluated for mechanical ventilation initiation, and four screening variables were recorded: intubation procedure note time, end-tidal CO_2_ recording time, peak inspiratory pressure (PIP) time, and positive end-expiratory pressure (PEEP) time. Given that the variables within the present study are only recorded in association with ventilation, their first appearance was recorded as the time point of ventilation initiation. It was necessary to record these variables because no reference standard exists for mechanical ventilation initiation when reviewed retrospectively. The variables analyzed in the current manuscript are translated into time points through the use of our institution’s ICU database [7]. The ventilator parameters are automatically downloaded within the database once the patient is connected to the ventilator. Thus, the mechanical ventilator parameters registered within the database are translated into time points in real-time. While the best outcome metric for a time point would clearly be prospective data capture, this was a retrospective study. PIP was the reference standard adopted in the present study because 1) the difference in mechanical ventilation parameters and intubation procedure note time varied by more than 60 minutes in 40% of the derivation subset, 2) end-tidal CO_2_ monitoring was present in roughly 15% of the derivation subset, and 3) PEEP was recorded with both noninvasive and invasive modes of mechanical ventilation vs PIP, which more accurately reflected invasive mechanical ventilation. The review included emergent and nonemergent intubations that occurred in the ICU. The review of non-emergent intubations was included as a systematic check to ensure the electronic search algorithm we published previously was accurate. Disagreements between the two reviewers were settled by a third reviewer (V.H.). The research team involved in manual data extraction was not aware of the automated electronic search strategy results.

### Automated electronic search strategy

The present retrospective study used the ICU data mart of METRIC [7]. The data mart contains such patient information as demographic characteristics, diagnoses, laboratory testing, flow sheets, clinical testing, and pathologic data gathered from various resources within the institution. It allows the application of search algorithms, such as the one described herein. Data is automatically captured from ventilators through the EMR. The ICU data mart has been validated and is reliable [7,8].

To develop the electronic search strategy, we first included such variables as intubation procedure note and end-tidal CO_2_ time to the ‘search query’ from our ICU database. Additional criteria consisted of mechanical ventilation parameters, such as PIP and PEEP times. The electronic search strategy was continuously refined through use of one variable as either intubation procedure note time, end-tidal CO_2_ time, PIP time, or PEEP time, or any combination of these variables. The variable PEEP was chosen for the automated electronic search rather than the PIP variable or the other parameters for the following reasons: 1) the PEEP variable had a nearly complete dataset unlike the PIP and end-tidal CO_2_ variables which was missing a significant portion of data within data mart and; 2) PEEP had a more accurate agreement with manual medical record review (using PIP as the reference standard) to within five minutes versus the intubation procedure note. Therefore, the final search algorithm consisted of PEEP with a five-minute restriction and the utilization of datasets with less than 15% missing data as an additional restriction. We choose an error of five minutes as the maximum acceptable error in order to precisely identify the start of mechanical ventilation. Parameters that differed more than five minutes may not accurately capture hemodynamic disturbances associated with emergent intubations.To validate the automated electronic search algorithm, an overall percent agreement plot was constructed by comparing the automated electronic search to the reference standard of comprehensive manual medical record review. When derived, the final electronic search algorithm consisting of PEEP was applied to an independent validation subset. A κ value was generated for both subsets. The automatic search strategy was done independently by a critical care research physician (R.K.). (For a flow chart of the process, see Figure 1.)

### Statistical analyses

Given that our outcome data are continuous, as time in minutes, an overall percent agreement between the search algorithm and manual medical record review was recorded. We agreed that a difference of five minutes between our EMR search strategy and the reference standard was acceptable. A Bland-Altman plot would have been ideal with the outcome variable; however, the outcome metric of interest was a time point, and thus an average of time when the majority of values differed by less than one minute did not make logical sense. Therefore, we report an overall percent agreement plot rather than a Bland-Altman plot.

Furthermore, we do not report on sensitivity and specificity of the current algorithm. Given that no gold standard exists and that the reference standard we adopted may be different in other institutions where, for example, end-tidal CO_2_ is continuously recorded, we felt it was misleading to report on sensitivity and specificity of the current algorithm. If we were to report on sensitivity and specificity, we would have then adopted the PIP variable as the gold standard. However, this may not be the consensus in the greater scientific community.

## Results

A total of 367 patients had intubations performed outside the ICU but were registered in the electronic system as requiring invasive mechanical ventilation. This effect was attributed to patients being intubated before their arrival in the ICU. The majority of patients (80%) were intubated in the operating room and transferred postoperatively to the ICU for ongoing mechanical ventilation. Missing data were noted for the majority of end-tidal CO_2_ recordings (>80%); this screening variable was excluded. Intubation procedure note time differed by more than 30 minutes from mechanical ventilation parameters in roughly 60% of the data. PEEP was present for both noninvasive and invasive mechanical ventilation, as opposed to PIP being present primarily for invasive mechanical ventilation only. Therefore, PIP was used as the reference standard on manual medical record review. Unfortunately, our data mart’s database lacked a large percentage of PIP measurements, but PEEP measurements were present within the entire database and therefore PEEP was used as the surrogate for PIP during the automated electronic search.

Manual data extraction was based on PIP as this variable was present during manual review of the patient’s electronic medical record. Although our ICU database contains a vast array of data, it does not contain each and every variable in a patient’s medical record and therefore, it may have some data in a format which can’t be queried, but is available for human data extraction.

**Figure 2:**
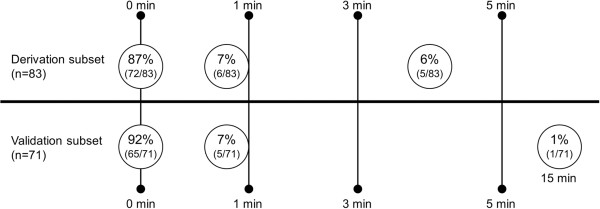
Overall percent agreement for derivation and validation subsets comparing the electronic search algorithm to manual comprehensive medical record review.

In the derivation subset, 83 patients were identified as requiring mechanical ventilation that began in the ICU and not in the emergency department or operating room by using our previously validated electronic search algorithm [1]. A perfect match, meaning a zero minute difference between the two variables, occurred in 72 patients (87%); the overall percent agreement to within one minute was 94% between manual medical record review with PIP and our search algorithm with data mart’s PEEP. Disagreement differed by no more than five minutes in the entire subset (n = 5). Overall agreement was found to be 87% (κ = 0.87).The search algorithm was further validated in 450 randomly selected patients seen in 2011. A total of 379 patients had intubations performed outside the ICU; 71 patients had initiation of mechanical ventilation after ICU admission, again using our previous electronic search algorithm. Perfect agreement was found in 65 patients (92%, zero minute difference), with 70 patients (99%) having agreement to within one minute. One patient had a difference of 15 minutes. Overall agreement was 92% (κ = 0.92). A plot of the overall percent agreement for the derivation and validation subsets is shown in Figure 2.

## Discussion

In the present study, we have shown a high degree of agreement between our search strategy using an institutional data mart system for the recognition of invasive mechanical ventilation initiation with the traditional method of manual medical record review. The search algorithm was both highly feasible and highly reliable. Agreement was perfect in more than 90% of medical records reviewed and differed by no more than one minute in 99% of medical records.

We accepted a disagreement to within five minutes. This five minute time point was established because we are primarily concerned with risk factors 60 minutes before and 60 minutes after endotracheal intubation. Five minutes on either side was believed to be reasonable, given the retrospective nature of the forthcoming study. PIP would have been more accurate for invasive mechanical ventilation. However, within our EMR, a large proportion of these data were not charted systematically and thus PEEP was used as a surrogate measure, which we believed was a reasonable alternative. When combined with the electronic note search strategy for emergent endotracheal intubation (published previously [1]), we are confident that the PEEP measurement represents a patient who at this moment underwent placement of an endotracheal tube emergently. Other mechanical ventilation variables, including, but not limited to, peak flow, respiratory rate, fraction of inhaled oxygen, and tidal volume, were either missing within the data mart system or believed to be inaccurate on manual medical record review.

The development of the two search algorithms was felt to be necessary before evaluating potential risk factors associated with hemodynamic disturbance during emergent endotracheal intubation in the ICU. Both the emergent and time-zero search strategies will allow rapid assessment of medical records and thereby save considerable time in reviewing the medical records. For example, the amount of time necessary to review the medical record for the establishment of initiation of invasive mechanical ventilation ranged from five minutes to approximately 20 minutes (per medical record) for the investigative team. Therefore, this search algorithm will substantially reduce the interval for establishing the timing of invasive mechanical ventilation.

The electronic search strategy used in this context has several limitations. First, we are operating under the assumption of accurate and timely data recording and note writing. With inconsistencies in the database, inaccurate results may have been recorded. However, inconsistencies in data are much less with automated search strategies than manual chart review [12]. In addition, the data mart’s database is monitored for integrity of data feed with periodic quality checks [7]. Second, we have used a database that is customary for our institution. This approach limits the applicability of these search strategies to areas with a similar database. With that said, the first requirement in any institution is access to electronic medical record data. Since we use routinely collected data, any other institution with access to their EMR database will be able to replicate this search strategy. Thus, an ‘Electronic Medical Records’ based database (warehouse/data mart) in any other institution would be able to replicate the results with the use of pertinent standard routinely collected clinical variables. In addition, given the adoption of the EMR in many large hospitalized systems, our approach is likely to become more generalizable. Third, data could have been entered in error or the database could have been corrupted [10]. This limitation is unlikely to be clinically significant because it accounts for only a small percentage of the database. Fourth, we chose a mechanical ventilation parameter that is known to be recorded for both invasive and noninvasive ventilation. Using the PIP parameter or additional respiratory parameters may have markedly improved the utility of our search algorithm. However, this approach was not practical in the present study because PIP (as well as other respiratory parameters) had a large proportion of missing data. Fifth, the algorithm may be considered retrospective in nature and not suited for real time. The data mart tool is a near–real-time database. Therefore, potential delays can affect rules when they are applied prospectively.

## Conclusions

The present study, along with the previously published study identifying emergent endotracheal intubations, illustrates how search algorithms can be used to accurately and rapidly navigate the EMR. The search strategy created for establishing time zero—the period of mechanical ventilation initiation in the ICU—resulted in a high degree of agreement with manual medical record review. Such algorithms can be used with any type of standard or customized software and are a reliable alternative to manual chart review.

The value in developing an electronic search algorithm relates both to cost and time savings in an ICU environment. Because of the severity of illness associated with critical care patients, data accumulation can be quite extensive. The large number of data points makes manual review of ICU patient records difficult. Although we focused on one element that is commonly performed in the ICU, the tools we utilized to arrive at our final conclusions can be adopted to retrospectively analyze many data points with both cost and time savings as compared to manual extraction with trained research personnel.

The present search algorithm along with the previously validated search strategy will allow analysis of possible risk factors associated with hemodynamic instability during emergent endotracheal intubation in the critically ill population. These search algorithms will rapidly reduce the time necessary to review medical records. Using our two search algorithms, we now will focus on assessing potential risk factors that may contribute to hemodynamic instability in the critically ill patient who has undergone emergent endotracheal intubation in the ICU.

## Abbreviations

EMR: Electronic medical record; ICU: Intensive care unit; METRIC: Multidisciplinary epidemiology and translational research in intensive care; PEEP: Positive end-expiratory pressure; PIP: Peak inspiratory pressure.

## Competing interests

The authors declare that they have no competing interests.

## Authors’ contributions

NS designed the study, performed data acquisition and data interpretation. RK designed the search algorithm and provided statistical analysis. VH designed the search algorithm. VM performed data acquisition. JO performed data acquisition. BP participated in data interpretation. All authors drafted the manuscript and/or revised it critically for important intellectual content and gave final approval of manuscript with all the accountability herein. All authors read and approved the final manuscript.

## Pre-publication history

The pre-publication history for this paper can be accessed here:

http://www.biomedcentral.com/1472-6947/14/55/prepub
